# The efficacy of γ-aminobutyric acid type A receptor (GABA _A_R) subtype-selective positive allosteric modulators in blocking tetramethylenedisulfotetramine (TETS)-induced seizure-like behavior in larval zebrafish with minimal sedation

**DOI:** 10.1016/j.taap.2021.115643

**Published:** 2021-07-13

**Authors:** Paige C. Mundy, Brandon Pressly, Dennis R. Carty, Bianca Yaghoobi, Heike Wulff, Pamela J. Lein

**Affiliations:** a Department of Molecular Biosciences, University of California, School of Veterinary Medicine, Davis, CA 95616, United States; b Department of Pharmacology, University of California, School of Medicine, Davis, CA 95616, United States

**Keywords:** Benzodiazepine, Chemical Threat Agent, GABA_A_R, Seizures, Tetramethylenedisulfotetramine, Zebrafish

## Abstract

The chemical threat agent tetramethylenedisulfotetramine (TETS) is a γ-aminobutyric acid type A receptor (GABA _A_R) antagonist that causes life threatening seizures. Currently, there is no specific antidote for TETS intoxication. TETS-induced seizures are typically treated with benzodiazepines, which function as nonselective positive allosteric modulators (PAMs) of synaptic GABA_A_Rs. The major target of TETS was recently identified as the GABA_A_R α2β3γ2 subtype in electrophysiological studies using recombinantly expressed receptor combinations. Here, we tested whether these *in vitro* findings translate *in vivo* by comparing the efficacy of GABA_A_R subunit-selective PAMs in reducing TETS-induced seizure behavior in larval zebrafish. We tested PAMs targeting α1, α2, α2/3/5, α6, ß2/3, ß1/2/3, and δ subunits and compared their efficacy to the benzodiazepine midazolam (MDZ). The data demonstrate that α2- and α6-selective PAMs (SL-651,498 and SB-205384, respectively) were effective at mitigating TETS-induced seizure-like behavior. Combinations of SB-205384 and MDZ or SL-651,498 and 2–261 (ß2/3-selective) mitigated TETS-induced seizure-like behavior at concentrations that did not elicit sedating effects in a photomotor behavioral assay, whereas MDZ alone caused sedation at the concentration required to stop seizure behavior. Isobologram analyses suggested that SB-205384 and MDZ interacted in an antagonistic fashion, while the effects of SL-651,498 and 2–261 were additive. These results further elucidate the molecular mechanism by which TETS induces seizures and provide mechanistic insight regarding specific countermeasures against this chemical convulsant.

## 1. Introduction

Tetramethylenedisulfotetramine (TETS) is considered a credible chemical threat agent that poses a serious public health risk to military personnel and civilians (Jett and Spriggs, 2020). Despite a worldwide ban on its production, TETS is still available on the black market as a rodenticide, and the weaponization of TETS together with accidental and suicidal poisonings has caused a significant number of human deaths (Li et al., 2014; Zhang et al., 2011; Zhou et al., 2011). Acute exposures can produce seizures that rapidly progress to *status epilepticus* in humans (Li et al., 2012; Zhang et al., 2011) and rodent models (Zolkowska et al., 2012), which can result in death if left untreated. Chronic epilepsy and memory deficits have been reported in survivors (Li et al., 2012).

Data from *in vitro* studies suggest that TETS induces seizures by antagonism of γ-aminobutyric acid type A receptors (GABA_A_R) (Lauková et al., 2020). GABA_A_Rs are heteropentameric ligand-gated chloride channels that exist in multiple subtype formations composed of various subunits, including α1–α6, β1–β3, γ1–γ3, δ, ε, π, θ, and ρ1–ρ3 (Olsen and Sieghart, 2009). The GABA_A_R subtype selectivity of TETS has been previously explored using whole-cell patch-clamp analysis of recombinantly expressed human synaptic and extrasynaptic GABA_A_Rs (Pressly et al., 2018). From these experiments, the major target of TETS was proposed to be α2β3γ2 (Pressly et al., 2018), which makes up 15–20% of the GABA_A_Rs in the mammalian brain (Rudolph and Knoflach, 2011). TETS inhibits α2β3γ2 receptors by binding to the non-competitive antagonist site in the channel pore (Pressly et al., 2020). All other tested GABA_A_Rs were significantly less sensitive except the α6β3γ2 isoform, which was blocked by sub-micromolar TETS concentrations (Pressly et al., 2018).

Currently, a medical countermeasure specific for TETS-induced seizures is not available (Jett and Spriggs, 2020). Intoxicated individuals are likely to be treated with benzodiazepines (Barrueto et al., 2003; Li et al., 2012), which are the standard of care for organophosphate-induced seizures. Classified as positive allosteric modulators (PAMs), benzodiazepines do not directly open GABA_A_Rs, but increase their sensitivity to the endogenous ligand GABA (Olsen, 2015). Benzodiazepines bind to a conserved site between multiple subtypes of GABA_A_R (Olsen, 2015; Schoch et al., 1985). Specifically, the α1, 2, 3, and 5 GABA subunits share a conserved histidine residue at the extracellular α - γ domain interface that serves as the binding site for benzodiazepines (Masiulis et al., 2019; Rudolph et al., 1999). The α4 and α6 subunits have an arginine at the corresponding position, rendering receptors containing these subunits insensitive to benzodiazepines (Olsen, 2015; Rudolph et al., 1999). While benzodiazepines are often effective as an immediate antiseizure treatment for GABA_A_R antagonist-induced seizures, latent neuropathology in these types of exposures is often not diminished by treatment (Lauková et al., 2020). Clinical reports describe incidences of recurrent seizures that are unresponsive even after initial successful mitigation with a benzodiazepine (Li et al., 2012; Whitlow et al., 2005). Further, adverse effects, including sedation or loss of consciousness (Ochoa and Kilgo, 2016), hypotension, and decreased heart rate (Kitajima et al., 2004), limit benzodiazepine dosing in the clinical setting. Given these shortcomings, there is an urgent need to elucidate the mechanism of TETS-induced seizures to develop more specific countermeasures than benzodiazepines.

Zebrafish express the full range of GABA_A_R subunits, including single homologs of α1, α3, α4, α5, β1, β2, β3, β4, ρ1, γ1, γ2, γ3, and δ subunits, and two paralogs for the α2 (gabra2a and gabra2b), α6 (gabrb6a and gabra6b), ρ2 (gabrr2a and gabrr2b), ρ3 (gabrr3a and gabrr3b), and π (gabrp and gabrz) subunits (Monesson-Olson et al., 2018). At the protein-encoding level, these receptors exhibit 60–98% homology with human GABA_A_R subunits (Klee et al., 2012). Our previous study describing an *in vivo* zebrafish (*Danio rerio*) model of TETS-induced toxicity showed that: 1) larval zebrafish responded to TETS with increased swimming activity, referred to herein as “seizure-like behavior”, that corresponded to stereotypic seizure behavior in zebrafish and to altered electrical field activity in the optic tectum that resembled electrographic seizure activity; and 2) this seizure-like behavior was attenuated by treatment with PAMs including MDZ (Bandara et al., 2020). Here, we used this zebrafish model to investigate the GABA_A_R subtype selectivity of TETS-induced seizurogenic behavior, using total distance moved as an endpoint that has been previously shown to correlate with seizure activity (Bandara et al., 2020; Baraban et al., 2005). The efficacy of MDZ in mitigating TETS-induced seizure-like behavior in larval zebrafish was compared to various compounds selective for GABA_A_R subtypes α2, α6, α1, α2/3/5, ß2/3, ß1/2/3, and δ (Table 1). In parallel we used whole-cell patch-clamp recordings of L929 cells expressing human GABA_A_ receptors α1, α2, α6, β3, and γ2L to confirm the published subtype selectivity of our most potent tool compounds: the α2-selective compounds NS11394 and SL-651,498, the α1-selective compound CL-218872, and the α6-selective compound SB-205384. A photomotor assay previously used to study chemical-induced hyper- and hypoactivity in zebrafish larvae (Dach et al., 2019) was used to assess sedative effects of MDZ and combinations of subtype-selective therapeutics. Alternating light-dark photomotor assays have been determined to better predict anticonvulsant-induced hypoactivity than other behavioral tests (Ogungbemi et al., 2019). The results from these studies show that combinations of PAMs can be effective in mitigating TETS-induced seizure-like behavior at concentrations that do not elicit sedative effects.

## 2. Methods

### 2.1. Zebrafish husbandry

Fish husbandry, spawning, and fish experiments were performed with the approval of the University of California, Davis, (UC Davis) Institutional Animal Care and Use Committee. All experiments complied with the NIH Guide for the Care and Use of Laboratory Animals (NIH Publications No. 8023, revised 1978; National Research Council (US), 2011) and ARRIVE guidelines (du Sert et al., 2020). Tropical 5D wild type zebrafish (obtained from Sinnhuber Aquatic Research Laboratory (SARL) at Oregon State University, Corvallis, OR, with subsequent generations raised at UC Davis) were maintained in a standalone aquatic flow-through system (Aquaneering, San Diego, CA). The water source, referred to as “system water”, was deionized water that was further purified by reverse osmosis (Reverse Osmosis System Model AAA-1005, Applied Membranes Inc., CA). Standard aquaculture conditions (Westerfield and ZFIN, 2000) were followed, including a 14:10 h light:dark cycle (~850 lx). System water was maintained at 28.5 ± 0.5 °C and supplemented with 20 g/L NaHCO_3_ to maintain a pH of 7.5 ± 0.3 and 40 g/L sea salt solution (Instant Ocean) to maintain conductivity at 650 ± 25 μS. System water was checked weekly to ensure for safe nitrite, nitrate, and ammonia levels. Adult fish were fed twice daily with commercially available GEMMA Micro 500 (Skretting, Salt Lake City, UT). Spawning was conducted overnight in false bottom chambers with sex separated females and males. The breeders were combined the next morning, and embryos subsequently collected and transferred to plastic petri dishes containing embryo medium (Westerfield and ZFIN, 2000). Embryos were kept in a light cycle-controlled (14:10 h light:dark) incubator at 28.5 °C until removed for experimental purposes. All experiments were conducted using 5 dpf larvae, a developmental stage at which the sex of the animal is not yet determined (Kossack and Draper, 2019).

### 2.2. Larval exposure to TETS and positive allosteric modulators (PAMs)

#### 2.2.1. Chemical source information

TETS (≥ 97% pure as determined by GC–MS) was synthesized in the laboratory of Dr. Bruce Hammock, UC Davis as previously described (Zhao et al., 2014). L-838417, PZ-II-029, TCS 1105 and SB-205384 were purchased from Tocris, Minneapolis, MN, USA. CL-218872 was purchased from Toronto Research Chemicals, Toronto, Ontario, Canada. SL-651498 was purchased from Axon Medchem, Reston, VA, USA. NS11394 was purchased from eNovation Chemicals, Bridgewater, NJ, USA. DS2, THIP (gabaxadol hydrochloride), and zolpidem were purchased from Sigma, St. Louis, MO, USA. Purity of all commercial chemicals based on the vendor supplied certificate of analysis was 98% or greater by HPLC. The compounds 2–261 and 2–314 were generously provided by Kelvin Gee (University of California, Irvine). TETS and PAMs were dissolved as 1000× stocks in 100% DMSO (Sigma, St. Louis, MO, USA) and kept in a −20 °C freezer until use. Further dilution to 2× sub-stocks was conducted using embryo medium immediately before exposures. Vehicle (DMSO) was kept below 0.3% for all exposure paradigms.

#### 2.2.2. Exposure paradigms

Zebrafish larvae were raised in petri dishes under normal rearing conditions (as described in section 2.1) until 4 days post-fertilization (dpf) when individual larvae chosen at random were placed into 96-well plates with 50 μL embryo medium and sealed with Parafilm^®^ to minimize evaporation. Following a 24-h acclimation period, 120 hpf larvae were treated with either TETS and subsequent PAMs (for seizure-like behavior assays) or PAMs only (for photomotor assays).

**Fig. 1.**
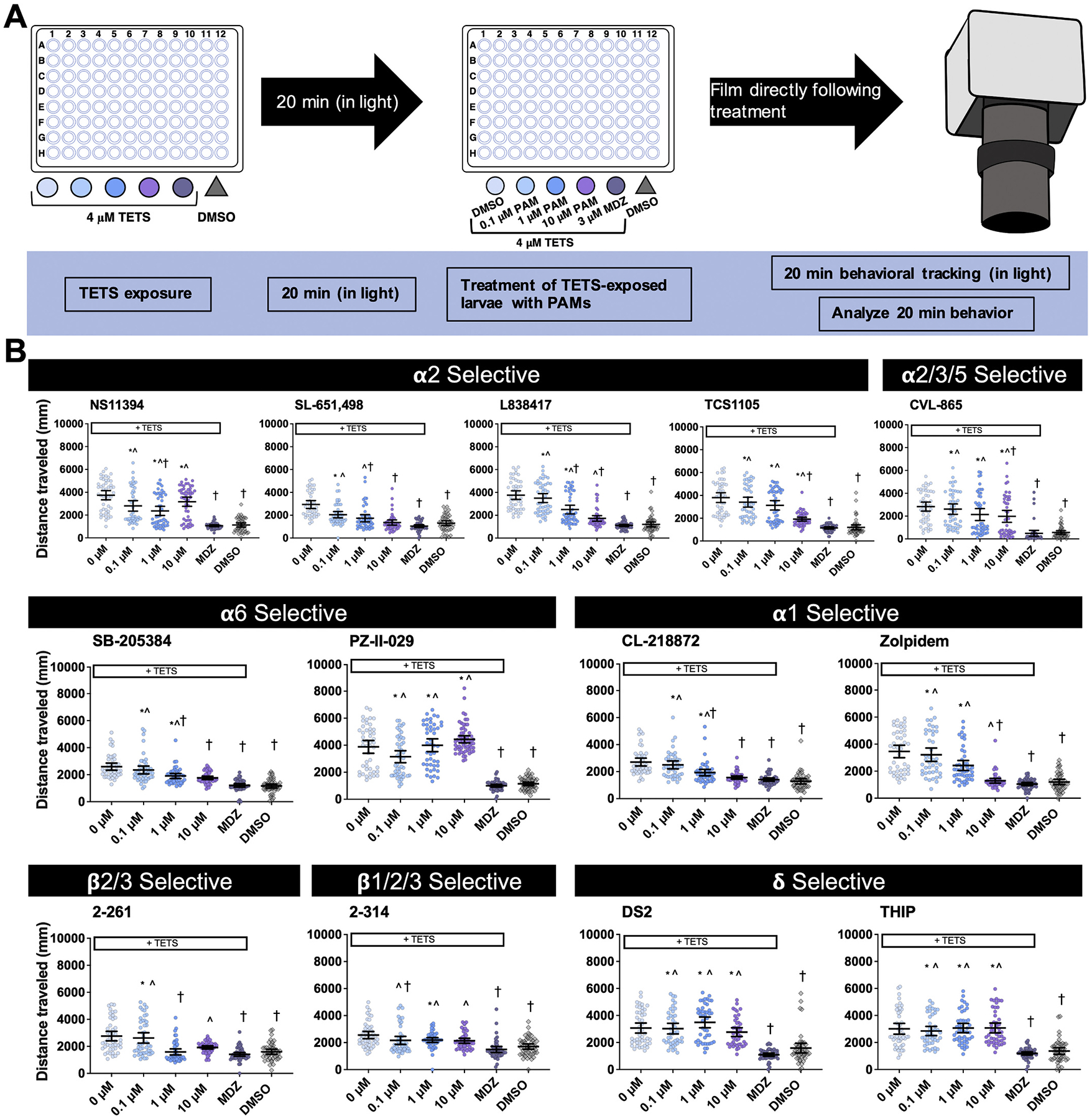
TETS exposure and zebrafish seizure-like behavior. (A) Experimental design of TETS-induced seizure-like behavior studies. (B) Movement of larval zebrafish (5 days post-fertilization) during the second 20 min tracking interval when co-exposed to TETS and GABA_A_R subtype-selective PAMs or MDZ. Individual dots represent individual larval replicates. Black bars represent mean ± 95% CI, *n* = 42–48 per group. Significant differences between groups determined using ANOVA with post-hoc Dunn’s multiple comparisons test. *Significantly different from DMSO controls at *p <* 0.05; ^significantly different from MDZ-treated larvae at *p <* 0.05; †significantly different from larvae exposed to TETS only at *p <* 0.05.

For TETS exposures, 120 hpf larvae were first treated with 50 μL of embryo medium containing 2× concentration of TETS or DMSO, resulting in a total of 100 μL aqueous solution per well (4 μM final concentration of TETS) and 20 min tracking was initiated after a ~5 min incubation period (Fig. 1). Following the first 20 min interval, PAMs were introduced *via* 50 μL of 3× final concentrations indicated in the figures, bringing the final volume to 150 μL. MDZ at a final concentration of 3 μM was included as a control in each experiment. For experiments investigating single PAMs, the final DMSO concentration for all wells was 0.17%, and for experiments investigating combinations of PAMs, the final DMSO concentration for all wells was 0.27%. Statistical analysis was conducted only between groups exposed to the same concentration of DMSO. We recognize measurable behavioral effects may occur with a difference of ±0.1% DMSO and thus have provided graphical and statistical comparison of controls (DMSO-only, TETS-only, and TETS +3 μM MDZ) across DMSO percentages (0.17% *vs.* 0.27%) in the supplementary material (Fig. S1). Concentrations of TETS (4 μM) and MDZ (3 μM) were chosen based on previous research (Bandara et al., 2020) indicating these concentrations consistently initiated and mitigated seizure-like activity, respectively, in Tropical 5D wild type zebrafish larvae.

For the photomotor behavioral tests, larvae were exposed to PAMs in the absence of TETS. As described above, zebrafish larvae were raised until 96 hpf and placed into 96-well plates with 50 μL embryo medium. Following a 24-h acclimation period, 120 hpf larvae were treated with 50 μL of embryo media containing 2× concentration of DMSO. Then 50 μL of EM containing 3× concentrations of MDZ, MDZ and SB-205384, or SL-651,498 and 2–261 were added, bringing the final volume to 150 μL per well. After 5 min of acclimation to the treatment, recording began. For experiments investigating single PAMs, the final DMSO concentration for all wells was 0.17%, and for experiments investigating combinations of PAMs (MDZ and SB-205384or SL-651,498 and 2–261) the final DMSO concentration for all wells was 0.27%.

All experiments were repeated in triplicate using larvae from separate spawns, with *n* = 16 or *n* = 12 larvae per treatment per replicate.

#### 2.2.3. Quantification of seizure-like behavior and photomotor behavior

Locomotor behavior was quantified using an automated DanioVision system (Noldus, Leesburg, VA). Before the initiation of exposures, the 120 hpf larvae in the 96-well plates containing embryo medium were placed into the DanioVision chamber (temperature maintained at 28.5 °C) to acclimate for ~5 min in full light (~1900 lx). Seizure-like behavior during the TETS exposure paradigm was recorded and quantified as the total distance moved (in mm) binned per 20 min. The seizure-like behavior assays were conducted in full light throughout the 20 min recording because it is known zebrafish larvae move less in the light, thus ensuring seizure-like behavior is occurring as a result of chemical exposure and not in response to a change in light. In the photomotor assay, zebrafish were exposed to 5 min of light, followed by 5 min of dark, 5 min of light, and a final 15 min of dark. Because zebrafish larvae at this developmental stage are known to freeze in the light and move in the dark (MacPhail et al., 2009), we leveraged the photomotor assay to assess potential sedating properties of PAMs. Locomotor behavior in the photomotor behavioral tests was quantified as the total distance moved (in mm) binned per 1 min for visualization, and binned per 5 min for statistical analysis. For transparency and clear representation of variability, graphical visualization of 5 min bins of individual larvae is shown in Fig. S2. These data were analyzed using EthoVisionXT 10 software (Noldus) and exported to Excel (Microsoft) and GraphPad Prism 8.4.3 (La Jolla, CA, USA) for statistical analyses.

#### 2.2.4. EC_50_ analysis

Compounds that were effective in reducing TETS-induced seizure-like behavior at 1 μM (NS11394, SL-651,498, CL-218872, SB-205384, and 2–261) or 1:3 combinations (1:3 MDZ:SB-205384, and 1:3 SL-651,498:2–261) were explored using a more graded concentration range in order to establish EC_50_ curves. Larval zebrafish were exposed to TETS at 4 μM, as described above, followed by addition of specific PAMs at 0.01, 0.03, 0.1, 0.3, 1, 3, and 10 μM. For the 1:3 combinations of PAMs, concentrations of 0.012, 0.04, 0.12, 0.4, 1.2, and 4 μM PAMs (described here as the total concentration of the mixture of both compounds) were tested. Each treatment was repeated in triplicate using larvae from three independent spawns, with *n* = 12–16 larvae per treatment per replicate. Concentrations were log transformed, and for each chemical, the average total distance traveled for all concentrations was normalized as a percentage, with 0% defined as the smallest mean and 100% defined as the largest mean.

### 2.3. Cell culture

The human GABA_A_R subunits α1, α2, α6, β3, and γ2L cloned into pcDNA3.1 expression vectors were a gift from Dr. Robert L. Macdonald (Vanderbilt University, Nashville, TN). L929 cells, a male mouse fibro-blast cell line (CCL-1), were obtained from American Type Culture Collection (Manassas, VA) and used for expressing all GABA_A_R subunits. L929 cells were cultured in Dulbecco’s modified Eagle’s medium (Lonza, Basel, Switzerland) supplemented with 10% fetal bovine serum, 100 U/mL penicillin and 100 mg/mL streptomycin (Invitrogen, ThermoFisher, Grand Island, NY) and maintained in humidified 95% air and 5% CO_2_ at 37 °C. L929 cells were transfected using FuGENE 6 (ThermoFisher) transfection reagent in Opti-MEM® reduced serum medium (Life Technologies, Benicia, CA) with an equal amount of each of the subunits (1:1:1) in combination with green fluorescent protein (GFP) expressed from the pEGFP-C1 vector (Invitrogen). The ratio of total cDNA to transfection reagent was 2:1. Cells were detached by trypsinization 48 h post-transfection, washed, and plated onto poly-l-lysine-coated glass coverslips. Transfected cells (GFP-expressing cells) were identified with an epifluorescence microscope used for electrophysiological whole-cell voltage-clamp studies.

### 2.4. Electrophysiological recordings

Whole-cell voltage-clamp recordings were performed at room temperature using an EPC-10 amplifier (HEKA Elektronik, Lambrecht, Germany). Cells were bathed in an external Ringer solution consisting of 160 mM NaCl, 4.5 mM KCl, 1 mM MgCl_2_, 2 mM CaCl_2_, 10 mM HEPES, pH 7.4, 308 mOsm. Recording electrodes were pulled from soda lime glass micro-hematocrit tubes (Kimble Chase, Rochester, NY) and fire-polished to resistances of 1.8–3 MΩ. Electrodes were filled with an internal solution consisting of 154 mM KCl, 2 mM CaCl_2_, 1 mM MgCl_2_, 10 mM HEPES and 10 mM EGTA, pH 7.2, 302 mOsm. Cells were voltage-clamped at 80 mV, and control currents were recorded under the local application of EC_10_ GABA for the respective GABA_A_R subtype for 5 s directly to the patch-clamped cell using an 8-channel pinch valve-controlled gravity-fed fast perfusion system (VC3–8xG system, ALA Scientific, Farmingdale, NY) positioned within 100 μm of the cell. GABA applications were followed by a 50-s wash with Ringer solution and then by a second application of EC_10_ GABA directly to cells after 1 min. The EC_10_ GABA for each studied receptor combination (1 μM for α1β3γ2, 2 μM for α2β3γ2, 0.3 μM for α6β3γ2) was previously determined (Pressly et al., 2018). PAMs were added to the chamber through a separate, syringe driven perfusion system and allowed to sit for 1 min on the cell before re-application of EC_10_ GABA. The area under the current curve (AUC) was measured with Patchmaster software (HEKA) and used to calculate the percent increase for each PAM compared to EC_10_ GABA.

### 2.5. Statistical analysis

**Fig. 2.**
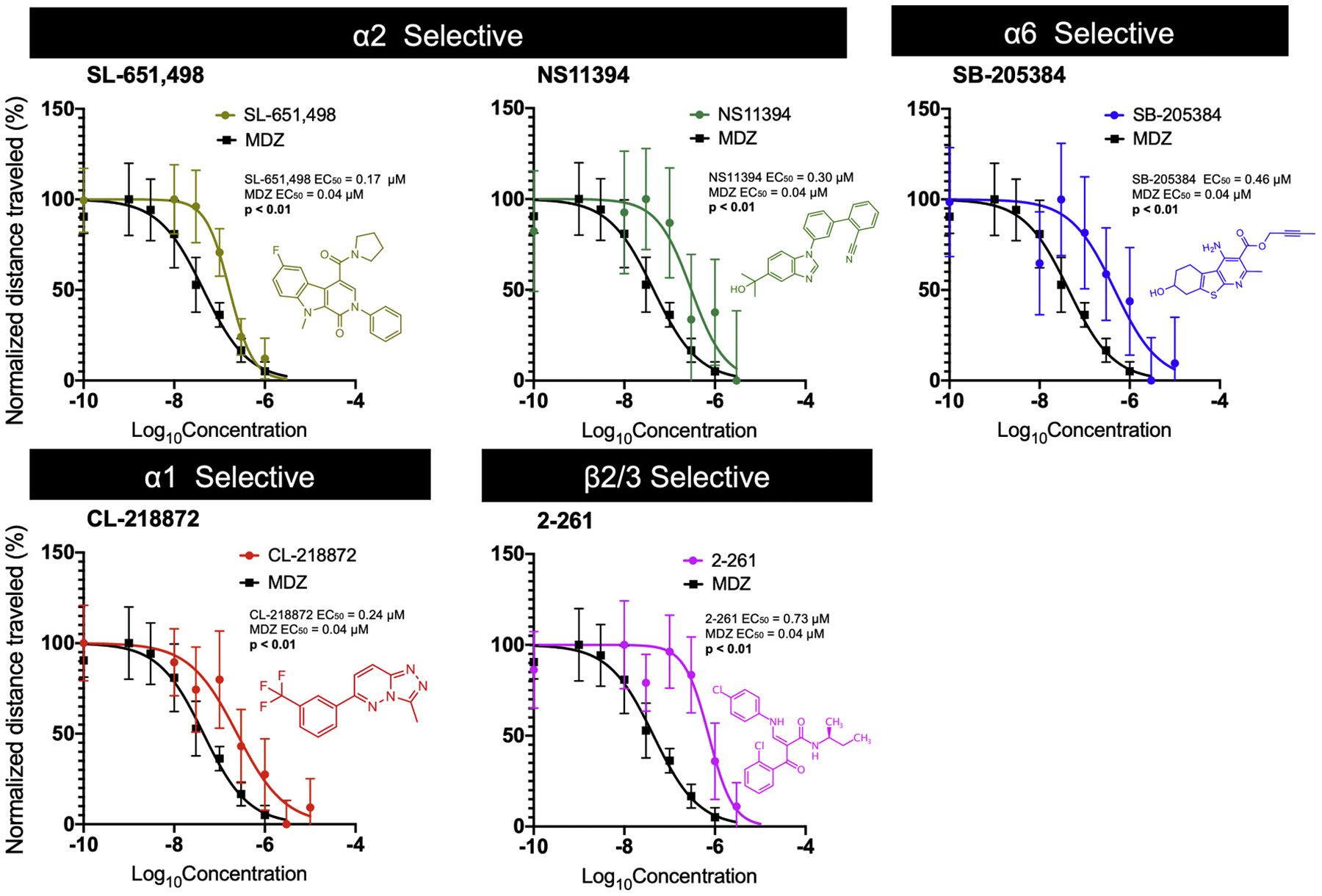
EC_50_ for efficacy of subtype-selective GABA_A_R PAMs in mitigating TETS-induced seizure behavior. Seizure-like behavior is shown as mean distance traveled normalized as a percentage, with 0% defined as the smallest mean and 100% defined as the largest mean. Individual points represent mean of all normalized larval fish movement (*n* = 32–36) for the second 20 min interval in the TETS exposure paradigm. Error bars represent ±95% CI. Curves were fit using nonlinear least-squares regression with no weighting. The logEC_50_ values between individual PAMs and MDZ were compared using extra sum-of-squares F Test (α < 0.05).

For all seizure behavior experiments, only the second 20 min interval was analyzed and graphed. The first 20 min interval was filmed for quality control purposes to ensure the TETS exposure induced the expected seizure-like behavior. Statistical testing was conducted using GraphPad Prism (version 8.4.3). To evaluate the seizure behavior data, a nonparametric Kruskal-Wallis analysis of variance (ANOVA) test was run. For post-hoc testing, Dunn’s multiple comparisons test (at α < 0.05) was used to compare all treatments to TETS-only exposed larvae, DMSO-only larvae, or MDZ (3 μM) treated larvae. For the photomotor assay, a nonparametric Kruskal-Wallis ANOVA test was run. For post-hoc testing, the mean of each treatment group for each 5 min total light or dark cycle was compared using Dunn’s multiple comparison’s test (α < 0.05) in comparison to DMSO-only larvae, or 3 μM MDZ treated larvae (Fig. 5). For the EC_50_ analysis, curves were fit to the transformed and normalized behavioral data using nonlinear least-squares regression with no weighting to evaluate the EC_50_’s in reference to behavioral data. The logEC_50_s between PAM and MDZ were compared using extra sum-of-squares F Test (α < 0.05) (Fig. 2).

For isobologram analysis, methods from (Huang et al., 2019) were followed. Full equations can be found in supplementary materials. In brief, the logEC_50_ values were generated for individual therapeutics (MDZ, SB-205384, SL-651,498, and 2–261) and drug combinations (1:3 MDZ:SB-205384 and 1:3 SL-651,498:2–261) using nonlinear least-square regression with no weighting. The theoretical additive EC_50_ values (theoretical EC_50_) were calculated for the drug combinations, and compared to the experimentally derived EC_50_ values (actual EC_50_) for each drug combination *via* an unpaired *t*-test (α < 0.05). Actual EC_50_ values that are significantly smaller than, larger than, or the same as the theoretical EC_50_ indicate synergism, antagonism, or additive interactions, respectively.

GraphPad descriptive statistics were used to determine the 95% confidence interval (CI) when comparing electrophysiological activity of compounds on subtypes. The concentration response curve of CL-218872 was fit with a four-parameter concentration-effect curve.

## 3. Results

### 3.1. Effect of GABA_A_R subtype-selective PAMs on TETS-induced seizure-like behavior

We tested GABA_A_R subtype-selective compounds to explore their efficacy in reducing TETS-induced seizures in larval zebrafish. At 5 dpf, zebrafish were exposed to 4 μM TETS, a concentration previously shown to induce seizure-like activity in Tropical 5D wildtype zebrafish larvae (Bandara et al., 2020) for 20 min, and subsequently treated with varying concentrations of GABA_A_R subtype-selective PAMs. Compounds selective for α2, α6, and β were most effective at reducing TETS-induced seizure-like behavior (Fig. 1). Of the α2-selective compounds, NS11394, SL-651,498, and L-838417 were effective at 1 μM, and TCS 1105 at 10 μM. SB-205384, an α6-selective compound, was effective at 1 μM. Of the α1-selective compounds tested, CL-218872 was effective at 1 μM and zolpidem at 10 μM. CVL-865, an α2/3/5-selective compound, was effective at 10 μM. Of the ß-selective compounds, the onset of effect occurred at a lower concentration for 2–314 (0.1 μM) than 2–261 (1 μM); however, the efficacy of 2–314 was not maintained at higher concentrations. Specifically, 2–314 plateaued between 0.1 and 10 μM, while 2–261 had solubility problems at 10 μM. The δ-selective compounds were not effective at any of the concentrations tested.

### 3.2. EC_50_ of individual PAMs

Compounds that were found to be effective in reducing TETS-induced seizure-like behavior at 1 μM (SL-651,498, NS11394, CL-218872, SB-205384, and 2–261) were further explored using a more graded concentration range in order to establish EC_50_ curves using the same assay as depicted in Fig. 1A. The EC_50_ of each GABA_A_R subtype-selective PAM was compared to the EC_50_ of MDZ since benzodiazepines are often used to treat TETS-induced seizures (Lauková et al., 2020). The EC_50_ for MDZ in our experimental design was 0.04 μM. The α2-selective compounds SL-651,498 and NS11394 had EC_50_ values of 0.17 and 0.30 μM, respectively, which were significantly higher than the EC_50_ of MDZ (Fig. 2). The α6- and α1-selective compounds SB-205384 and CL-218872 exhibited EC_50_ values of 0.46 and 0.24 μM, respectively, which were also significantly higher than the EC_50_ of MDZ. The EC_50_ value of the ß2/3-selective compound 2–261 (0.73 μM) was much higher than that of MDZ.

### 3.3. Electrophysiology results

**Fig. 3.**
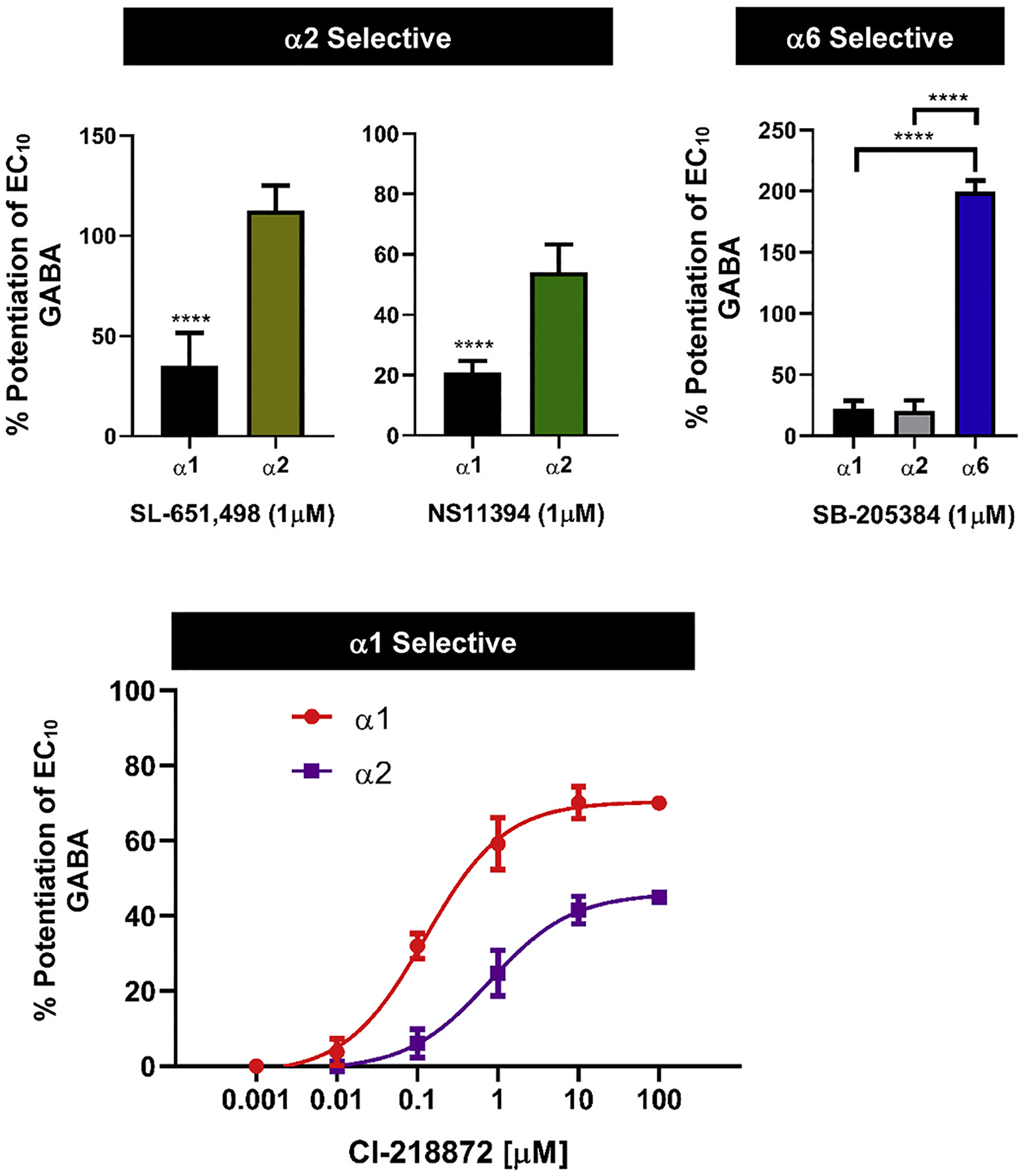
PAM subtype selectivity as determined using whole-cell patch-clamp. These graphs show percent potentiation of EC_10_ GABA in whole-cell voltage-clamp recordings in mouse L292 cells expressing the human GABA_A_ receptors α1β3γ2L, α2β3γ2L or α6β3γ2L. Bar graphs represent mean potentiation, and error bars represent ±95% CI, n = 4–8 cells for each graph or data point, ***p < 0.05 in *t-*test.

Previously published work on a number of our tool compounds was limited to binding assays and some of our behavioral results were not in keeping with the published selectivity profile of the α2- and α6-selective compounds. Thus, we felt that it was necessary to re-evaluate the GABA_A_R subtype selectivity of the less well-characterized tool compounds in our screen. We therefore performed whole-cell voltage-clamp recordings on L929 cells expressing the human GABA_A_R α1β3γ2L, α2β3γ2L or α6β3γ2L in order to test the respective compounds for their ability to enhance GABA elicited currents at the GABA EC_10_, where PAM activity is usually evaluated in electrophysiological assays to create large assay windows. Both SL-651,498 and NS11394 were found to be α2-selective in keeping with previously published work (Griebel et al., 2003; Mirza et al., 2008). SL-651,498 enhanced the activity of GABA on α1β3γ2L by 35.1% (CI 17.8–52.) and on α2β3γ2L by 112.5% (CI 92.7–132.4%). NS11394 enhanced the effect of GABA on α1β3γ2L by 20.8% (CI 14.0–25.6%) and on α2β3γ2L by 54.2% (CI 42.9–65.4%) (Fig. 3). The α6-selective compound SB-205384 had low activity on both α1β3γ2L and α2β3γ2L with 22.3% (CI 16.2–28.4%) and 20.7% (CI 11.9–29.4%) enhancement, respectively, that was eclipsed by its 199.6% (CI 188.5–210.7) potentiation on α6β3γ2L. The α1-selective compound CL-218872 was found to have more activity on α1, with an EC_50_ of 125 nM (CI 87.5–178.9 nM) and an E_max_ of 70.4%, than on α2, with an EC_50_ of 781.3 nM (CI 436.9–1353 nM) and an E_max_ of 46.0%, showing far less selectivity between α1β3γ2L and α2β3γ2L than previous binding assays had suggested (Atack et al., 1999).

### 3.4. Effect of PAM mixtures on TETS-induced seizure-like behavior and photomotor behavior

**Fig. 4.**
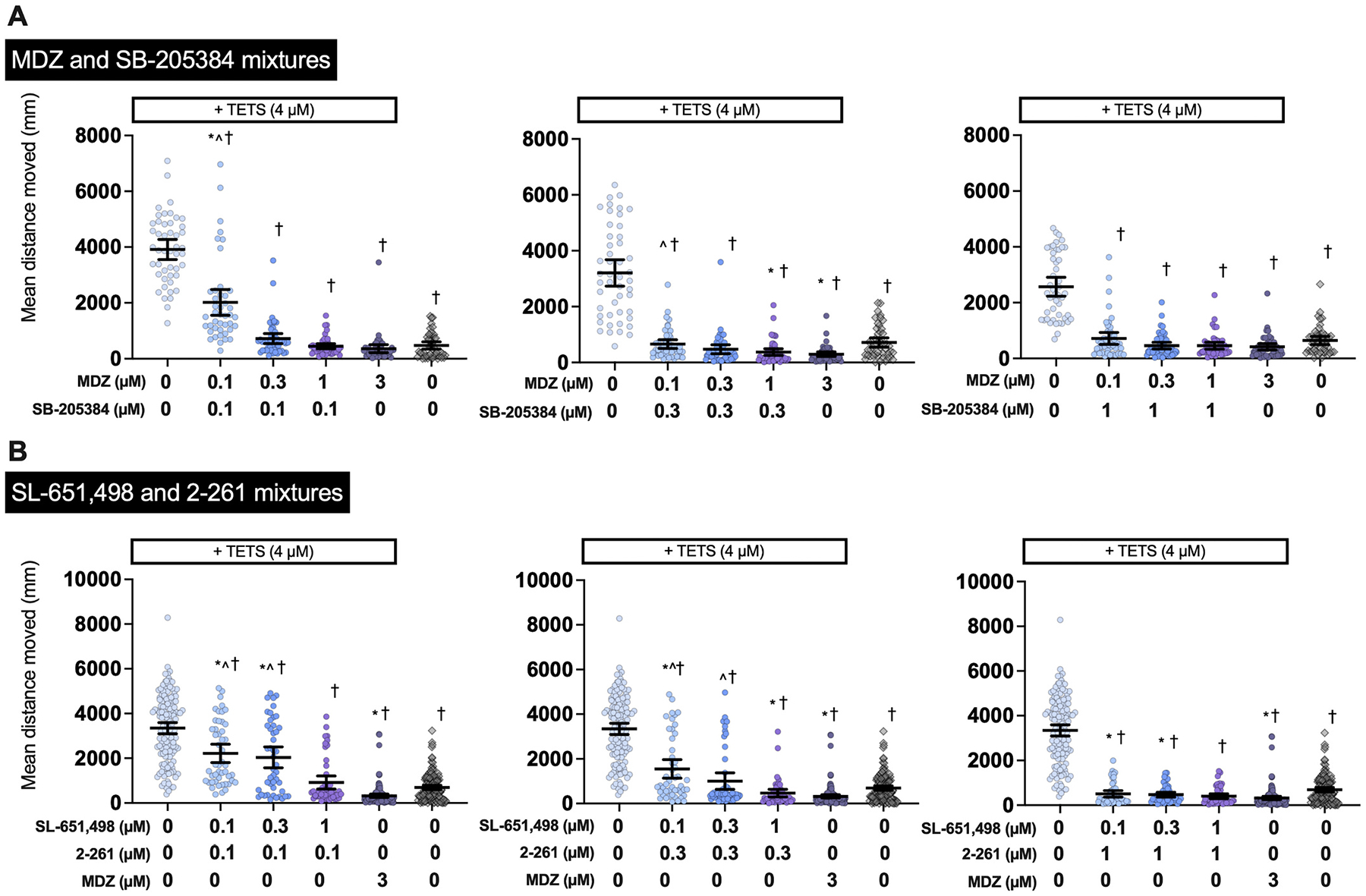
TETS seizure mitigation *via* therapeutic mixtures. Movement of 5 dpf larval zebrafish when co-exposed to TETS and mixtures GABA_A_R subtype-selective compounds A) MDZ and SB-205384, or B) SL-651,498 and 2–261, both including 3 μM MDZ control. Individual dots represent individual larva. Black bars represent mean ± 95% CI, *n* = 43–48. Significant differences between groups determined using ANOVA with post-hoc Dunn’s multiple comparisons test. *Significantly different from DMSO controls at *p <* 0.05; ^significantly different from MDZ-treated larvae at *p <* 0.05; †significantly different from larvae exposed to TETS only at *p <* 0.05.

After identifying the GABA_A_R subtype-selective compounds most effective at mitigating TETS-induced seizure-like behavior (SB-205384, SL-651,498, and 2–261), we assessed the efficacy of PAM combinations, with a focus on compounds selective for the α2, α6, and ß2/3 subunits. The lowest combination concentration tested of MDZ (nonselective) and SB-205384 (α6-selective), 0.1 μM and 0.1 μM respectively, was observed to significantly reduce TETS-induced seizure-like behavior in comparison to TETS-only exposed larvae (Fig. 4A); however, this combination was significantly different in comparison to DMSO-only control. A mixture of 0.1 μM MDZ and 0.3 μM SB-205384 reduced TETS-induced seizure-like behavior in comparison to TETS-only exposed larvae, and was not significantly different from DMSO-only control larvae (Fig. 4A). Combination concentrations of 0.1 and 0.1 μM, 0.1 and 0.3 μM, and 0.3 and 0.1 μM of SL-651,498 (α2-selective) and 2–261 (ß2/3-selective), respectively, significantly reduced TETS-induced seizure-like behavior in comparison to TETS-only exposed larvae (Fig. 4B). The results from this experiment informed the concentrations tested in the photomotor assay.

**Fig. 5.**
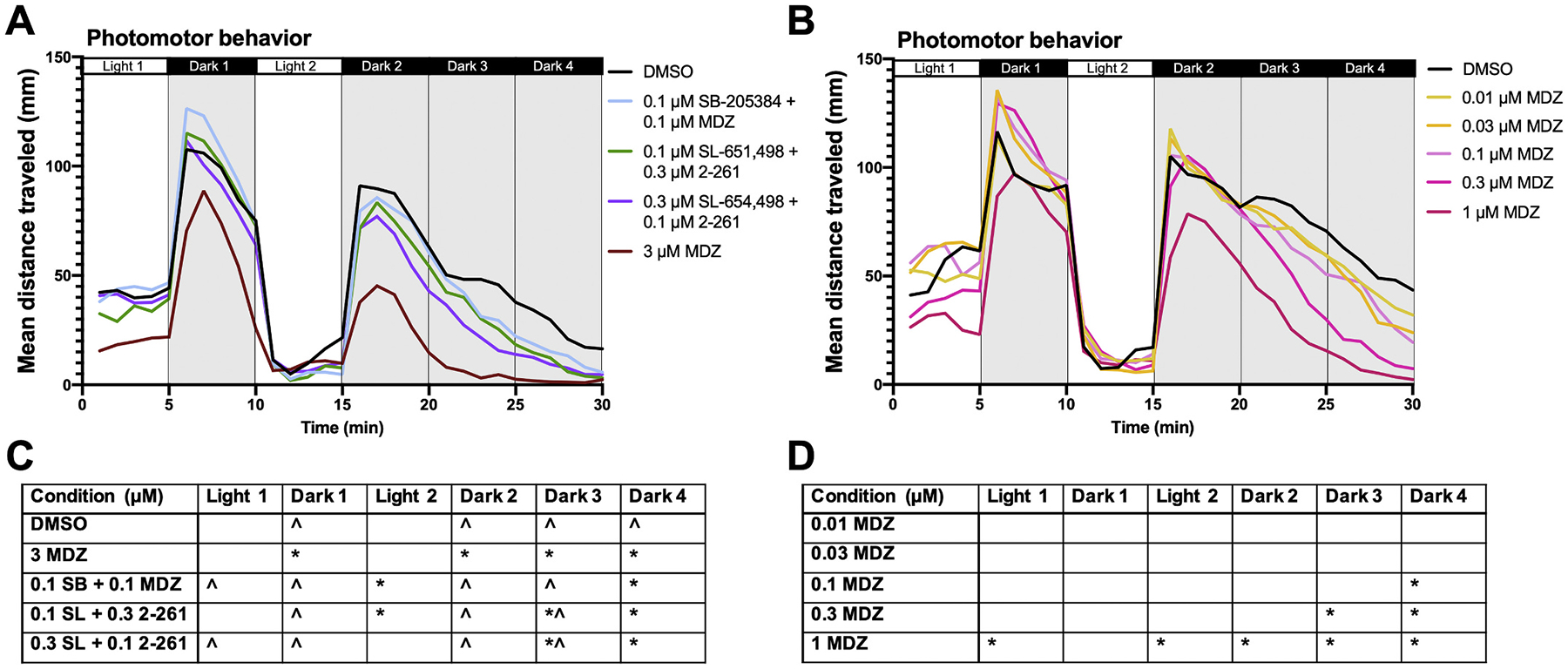
Photomotor behavior of larval zebrafish exposed to PAMS in the absence of TETS. Movement of 5 dpf larval zebrafish during a 30-min photomotor behavioral test after exposure to: A) combinations of PAMs or B) increasing concentrations of MDZ. Lines represent the mean total distance moved (mm) of each treatment group of larvae (n = 43–48). C and D) Summary of statistical analyses using ANOVA with Dunn’s multiple comparison’s test. *Significantly different from DMSO controls at *p <* 0.05; ^significantly different from larvae treated with 3 μM MDZ at *p <* 0.05.

To determine the potential for sedative effects of the PAM combinations that were most effective at mitigating TETS-induced seizure-like behavior, we measured larval responses to these PAMs in the absence of TETS using a photomotor assay. At this developmental stage, zebrafish larvae are known to freeze in the light and move in the dark (MacPhail et al., 2009), thus, we compared the total distance moved of the treated larvae to DMSO-only control larvae in each light or dark cycle. We considered compound concentrations and/or combinations that elicited hypoactivity during at least two periods that occur after a light-change stimulus (Dark 1, Light 2, or Dark 2) to be representative of sedation. Larvae exposed to a combination of 0.1 and 0.1 μM SB-205384 and MDZ moved approximately 2× less during Light 2 and Dark 4 in comparison to DMSO (Fig. 5A). Both combinations of SL-651,498 and 2–261 (0.1 and 0.3 μM, and *vice versa*) moved less in comparison to DMSO during Dark 3 (by ~1.5× and ~2×, respectively) and Dark 4 (by ~3× for both combinations). When exposed to 3 μM MDZ, movement was significantly decreased during all dark periods by ~1.5× (Dark 1), ~2.5× (Dark 2), ~9.5× (Dark 3), and ~15.4× (Dark 4). Considering MDZ is the current standard treatment for acute TETS intoxication, we also performed a concentration-dependent photomotor assay to determine at which concentration the sedative effect of MDZ begins. At 1 μM MDZ, mean distance moved is significantly less than DMSO control larvae during all light cycles except for Dark 1 by ~2× (Light 1), ~1.4× (Dark 2), ~1.1× (Light 2), ~2.8× (Dark 3), and ~9× (Dark 4) (Fig. 5B).

### 3.5. Isobologram analysis

Because the combination of MDZ and SB-205384 and of SL-651,498 and 2–261 were effective in mitigating TETS-induced seizure-like behavior (Fig. 4), we performed an isobologram analyses to determine whether these were additive, antagonistic, or synergistic interactions. To do this we obtained a concentration-effect curve of the compounds at a 1:3 ratio, established the EC_50_ of the combination and compared it to the theoretically calculated EC_50_ to determine synergy, antagonism, or additivity of the mixture.

**Fig. 6.**
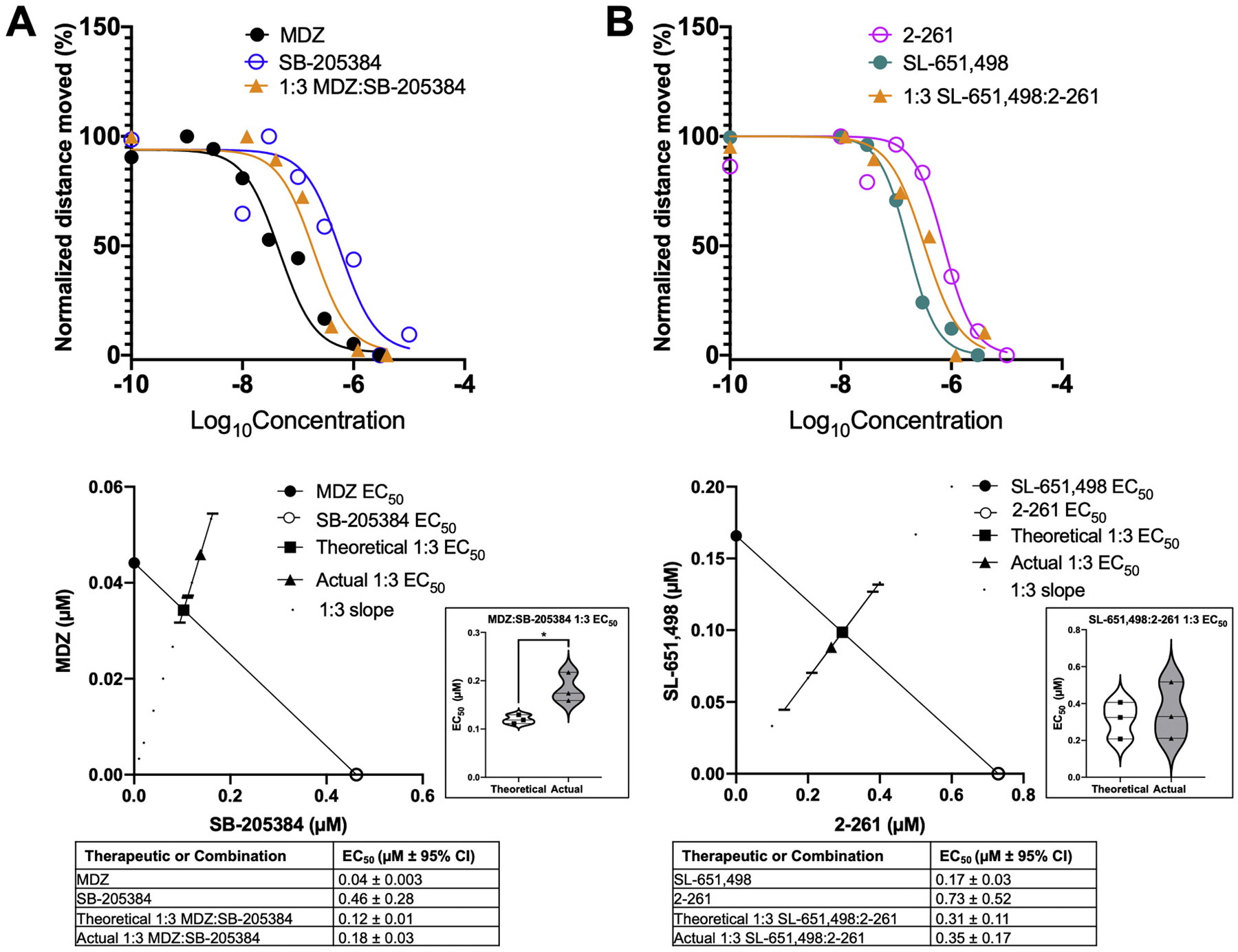
Isobologram analysis of PAM combinations. Movement of 5 dpf larval zebrafish following administration of A) 1:3 MDZ:SB-205384 or B) 1:3 SL-651,498:2–261 following exposure to 4 μM TETS for 20 min. Concentration-effect curves at the top show individual points representative of the mean of all normalized larval fish movement (*n* = 36–48) for the second 20 min interval in the TETS exposure paradigm. Curves were fit using nonlinear least-squares regression with no weighting. Isobolograms on the bottom are contour plots showing the calculated EC_50_ of each PAM (open or closed circle), connected by the line of additivity. Squares represent the mean of theoretical EC_50_ of a 1:3 combination if the mixtures were purely additive (n = 3), and triangles represent the mean of measured EC_50_ of a 1:3 combination of mixtures (n = 3). Error bars represent ±95% CI. Violin plots show the comparison of theoretical and actual EC_50_s (each point calculated from one replicate) using an unpaired t-test, *p < 0.05 (n = 3).

From these data, the 1:3 combination of MDZ: SB-205384 is suggested to be antagonistic in nature, considering the actual EC_50_ (0.18 ± 0.03 μM) is significantly higher than the theoretical EC_50_ (0.12 ± 0.01 μM) (Fig. 6A). There was no statistical difference between the actual EC_50_ (0.35 ± 0.17 μM) and theoretical EC_50_ (0.31 ± 0.11 μM) of the 1:3 mixture of SL-651,498: 2–261, indicating the compounds act in an additive fashion at these concentrations (Fig. 6B).

## 4. Discussion

Due to their importance in determining neuronal excitability, the roles of different GABA_A_R subunits in the pharmacology of both proconvulsants and antiseizure compounds have been extensively investigated (Olsen and Sieghart, 2009; Sills and Rogawski, 2020). Here, we explored if the previously determined subtype selectivity of the proconvulsant TETS (Pressly et al., 2018) could predict physiological outcomes *in vivo* when treating zebrafish larva with GABA_A_R subtype-selective PAMs following TETS exposure. We compared the current standard treatment for TETS intoxication, MDZ, to compounds selective for specific GABA_A_R subtypes. MDZ is a GABA_A_R PAM that non-selectively potentiates all synaptic GABA_A_Rs but does not affect α6 and α4-containing extrasynaptic receptors. Our data shows that α2-selective compounds, such as NS11394 and SL-651498 and the α6-selective PAM, SB-205384, are effective in decreasing TETS-induced seizure-like behavior in zebrafish. However, these compounds were not quite as potent as MDZ in terms of EC_50_.

The ability of CL-218872, an α1-selective compound, to effectively decrease seizure-like behavior at 1 μM following TETS intoxication was a surprising result considering it is only a partial agonist for the benzodiazepine site (Skerritt and Macdonald, 1984; Wingrove et al., 2002) and therefore should be less active than MDZ. However, follow-up studies indicated that the EC_50_ of CL-218872 was six-fold higher than MDZ. This is consistent with our previous work showing that TETS inhibits α_1_β_X_γ_2_ containing GABA_A_Rs about 10-fold less potently than α2 and α6 containing receptors (Pressly et al., 2018). Although the compound has previously been reported as being primarily an α1 GABA_A_R PAM (Skerritt and Macdonald, 1984), we found that it still maintains considerable functional activity on the α2 subunit in electrophysiology. This substantial activity on α2 subunits together with potentially increased penetration into the zebrafish tissue could explain the compound’s unexpected efficacy in this model.

Regarding the contribution of the ß subunits, our results suggest that PAMs targeting ß2 and ß3 can antagonize TETS’ activity as demonstrated by the high efficacy of 2–261 (ß 2/3-selective) at 1 μM. Interestingly the broadly ß-targeted 2–314 (ß1/2/3-selective) lacked efficacy. One potential explanation is that 2–314 penetrates poorly into the zebrafish brain tissue. An important caveat of this study is that we did not measure compound concentrations in fish tissues, therefore, we cannot rule out the possibility that differences in potency between compounds reflects differences in their uptake into the zebrafish brain (Tal et al., 2020). Lipophilic compounds, such as midazolam and most compounds investigated in this study, that exhibit logP values in the range of 2–3.5 are typically taken up well into zebrafish tissue. This is supported by a previous study in which we measured midazolam and diazepam concentrations in zebrafish tissue and water, and reported the body and brain had 5-fold or 10-fold higher concentrations than water (Bandara et al., 2020).

Questions remain regarding the role of the δ subunit. We attempted to evaluate extra-synaptic receptors using the limited number of δ-selective compounds currently available; however, due to solubility issues, we potentially under-dosed larvae. DS2 is a relatively polar compound with poor brain penetration in both mice and rats (Jensen et al., 2013) and therefore presumably also in zebrafish. THIP only maintains δ selectivity at low concentrations and acts as a conventional nonselective agonist at higher concentrations (Meera et al., 2011). To circumvent these issues, more potent δ-selective compounds are needed.

Our evaluation of combinations indicated that 0.1 μM SB-205384 (α6-selective) combined with 0.1 μM MDZ significantly mitigated TETS-induced seizure-like behavior. The efficacy of reduced concentrations of MDZ when combined with an α6-selective PAM is a promising finding considering the dose limitations of benzodiazepines due to adverse effects such as sedation. Leveraging the fact that zebrafish larvae naturally move more during the dark periods and remain relatively still during the light periods, we used a photomotor assay as a proxy for testing the sedative effects of PAM combinations. Although the neural circuit for behavior in the dark periods of photomotor assays has yet to be fully defined, it has been determined that anticonvulsants that display sedative properties cause hypoactivity during the dark periods of alternating light-dark photomotor assays (Ogungbemi et al., 2019). A combination of 0.1 μM SB-205384 and 0.1 μM MDZ showed no obvious sedating effects, whereas concentrations of MDZ ≥1 μM caused clear evidence of sedation. Although MDZ remains the most effective PAM tested in this study in terms of EC_50_ for terminating TETS-induced seizure-like behavior, our data show that combinations of SL-651,498 (α2-selective) and 2–261 (β2/3-selective) can be equally effective. These results corroborate our previous findings that TETS preferentially inhibits α2β3 containing GABA_A_ receptors. Further, combinations of 0.1 μM SL-651,498 and 0.3 μM 2–261 induced sedative behavior only in Dark 3 and 4 of the photomotor assay, as opposed to all dark periods, suggesting these combinations are less sedating than MDZ.

The 1:3 combination of MDZ and SB-205384 was found to act in an antagonistic fashion. Midazolam has been found to interact competitively with other GABA_A_R agonists. For example, flumazenil, an imidazobenzodiazepine that binds at the interface of α (nonselectively) and γ2 (Kucken et al., 2003) is used clinically to treat benzodiazepine overdoses (Seger, 2004). Heidelberg et al. (2013) reports that SB-205384 most likely does not work *via* the same site as flumazenil by testing the modulation of α3ß3γ2 by flumazenil and SB-205384 *in vitro*. It is also reported that SB-205384 maintains some activity on α5 (Heidelberg et al., 2013). Considering these two observations, the antagonism observed in this study could be occurring at the α5-γ2 interface; however, further testing is needed to confirm this. The 1:3 combination of SL-651,498 and 2–261 was found to act in an additive fashion. Considering SL-651,498 is proposed to bind to the benzodiazepine site (Griebel et al., 2003), the additive action could be mediated by the SL-651,498 activity on the α2–γ2 interface and by 2–261 activity on β2/3 subunits. An additive interaction could be beneficial in that the SL-651,498 and 2–261 combination could potentially alleviate the adverse effects associated with the use of high concentrations of benzodiazepines. This suggests a need to further investigate additional combinations of α2 and β2/3 PAMs.

In summary, our data provide insight into the *in vivo* mechanisms of TETS-induced seizures and suggest potential therapeutic strategies that involve the use of subunit-selective PAMs as adjuncts to MDZ for treatment of TETS poisonings, specifically combinations including α6-, α2- and ß2/3-selective PAMs. Zebrafish appear to be an appropriate *in vivo* model for continuation of this research as subunits α6b, β3, and γ2 are known to be expressed in the optic tectum and cerebellum in adult zebrafish, and it is hypothesized that an α6bβ3γ2 combination is possible (Cocco et al., 2017). Similarly, larval zebrafish have been found to express α1, α2a, α6b, β3, and γ2 in the optic tectum at 5 dpf (Monesson-Olson et al., 2018; Sadamitsu et al., 2021). *In vivo* experimentation using GABA_A_R subunit targeted knock-outs is a possible avenue to further confirm the role of GABA_A_R subunits α2 and α6 in TETS toxicity. Further, EEG experiments will be performed using zebrafish larvae to confirm the anti-seizure activity of promising therapeutics/combinations before moving to mammalian models. Considering that conventional benzodiazepines have adverse effects and are not always effective in terminating TETS-induced seizures, discovering alternative treatments is important. The compounds in this study found to be most efficacious in mitigating seizure behavior (SL-651,498, 2–261, and SB-205384) have promising therapeutic potential for treating TETS-induced seizures in mammals. All three compounds (SL-651,498, 2–261, and SB-205384) have previously been used in mammalian models and shown to have anxiolytic properties, with SL-651,498 and 2–261 causing minimal ataxia (Gee et al., 2010; Licata et al., 2005; Navarro et al., 2006). Better understanding of TETS toxicity will allow the direct targeting of necessary subunits, leading to safer and more effective treatments. Thus, therapeutic use of combinations that maintain anti-seizure efficacy while minimizing sedative effects are advantageous to pursue.

## Supplementary Material

Mundy et al., 2021, supplementary material

## Figures and Tables

**Table 1:** GABA_A_R subtype selectivity of PAMs evaluated in this study.

Therapeutic or Combination	GABA_A_R Subunit Selectivity	Reference	Reported electrophysiology findings in this study	Concentration at which TETS-induced seizure behavior is mitigated	Concentration at which sedation in photomotor assay occurs
Midazolam (MDZ)	Nonselective	(Sigel and Steinmann, 2012)	–	3 μM	1 μM
NS11394	α2	(Mirza et al., 2008)	α2-selective	1 μM / NA (solubility issues)	–
SL-651,498	α2	(Griebel et al., 2003)	α2-selective	1 μM	–
L-838417	α2	(McKernan et al., 2000)	–	1 μM	–
TCS 1105	α2	(Taliani et al., 2009)	–	10 μM	–
CVL-865	α2/3/5	(van Amerongen et al., 2019)	–	10 μM	–
SB-205384	α6	(Heidelberg et al., 2013)	α6-selective	1 μM	–
PZ-II-029	α6	(Varagic et al., 2013)	–	NA	–
CL-218872	α1	(Skerritt and Macdonald, 1984)	α1-selective with some activity at α2	1 μM	–
Zolpidem	α1	(Che Has et al., 2016)	–	10 μM	–
2–261	ß2/3	(Sieghart and Savic, 2018)	–	1 μM	–
2–314	ß1/2/3	(Gee et al., 2010)	–	0.1 μM / NA (solubility issues)	–
DS2	δ	(Wafford et al., 2009)	–	NA	–
THIP	δ	(Krogsgaard-Larsen et al., 2004)	–	NA	–
Combination of MDZ: SB-205384	Nonselective, α6	–	–	0.1 μM: 0.1 μM	NA
Combination of SL-651,498: 2–261	α2, ß2/3	–	–	0.1 μM: 0.1 μM	NA
