# Supplementary material for: The efficacy of γ-aminobutyric acid type A receptor (GABA AR) subtype-selective positive allosteric modulators in blocking tetramethylenedisulfotetramine (TETS)-induced seizure-like behavior in larval zebrafish with minimal sedation
Source: Toxicol Appl Pharmacol. Author manuscript; Available in PMC 2021 Oct 13. (PMC8514104; doi:10.1016/j.taap.2021.115643)
Supplement: Mundy et al., 2021, supplementary material [file NIHMS1745152-supplement-Mundy_et_al___2021__supplementary_material.docx]

**The efficacy of** γ**-aminobutyric acid type A receptor (GABA _A_R) subtype selective positive allosteric modulators in blocking tetramethylenedisulfotetramine (TETS)-induced seizure-like behavior in larval zebrafish with minimal sedation**

**Supplementary Material**

**SUPPLEMENTARY METHODS**

The equations for isobologram analysis were followed according to (Huang et al., 2019).

Potency ratio (R_B_) of compounds A and B were calculated as follows:

R_B_ = EC_50,A_ / EC_50,B_

The potency ratio was used to calculate the theoretical EC50s, using the equation below:

Theoretical EC_50_ = EC_50,A_ / (P_A_ + R_B_ X P_B_),

Where P_A_ and P_B_ are the proportions of compounds A and B in the mixture.

**SUPPLEMENTARY FIGURES**

**Figure S1. DMSO percentage comparison between experiments.** All data from larval zebrafish exposed to DMSO, TETS-only, or TETS + 3µM MDZ from Figures 1 and 4 were combined and analyzed to evaluate the effect of 0.17% DMSO vs. 0.27% DMSO on controls. Individual dots represent individual larva. Black bars represent mean ± 95% CI, n = 247 - 553. Significant differences between groups determined using ANOVA with post-hoc Dunn’s multiple comparisons test. *Significantly different p<0.05

**
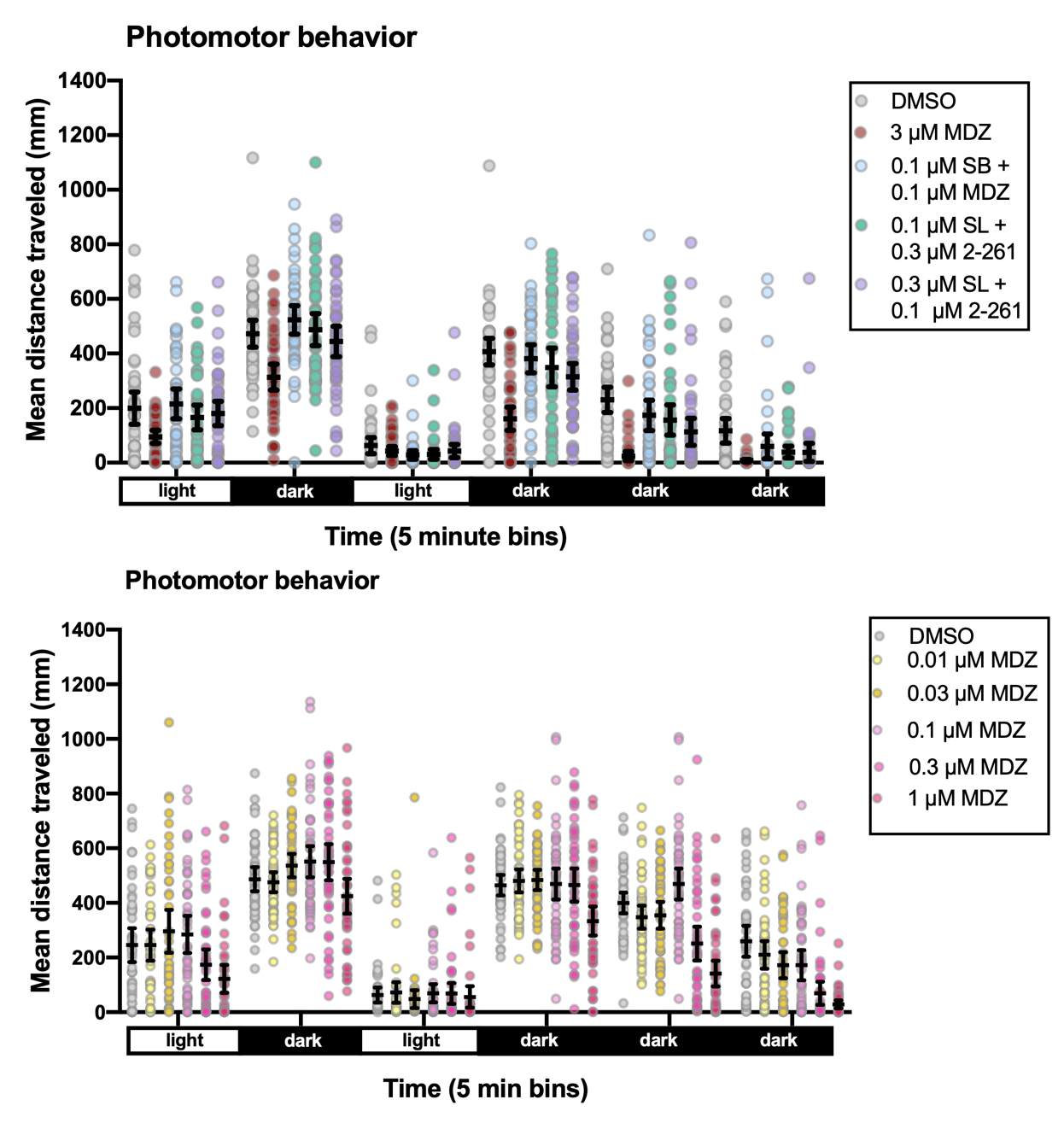
**

**Figure S2. Photomotor behavior during exposure to therapeutics.** Larval zebrafish (5 dpf) movement during a 30-minute photomotor behavioral test exposed to either mixtures of therapeutics or increasing doses of MDZ. Individual dots represent individual larval replicates (the mean of total distance moved over the 5 min light or dark period). Black bars represent mean ± 95% CI (n = 43 - 48).
